# Pelvic cardiovascular magnetic resonance venography: venous changes with patient position and hydration status

**DOI:** 10.1186/s12968-018-0503-6

**Published:** 2019-01-03

**Authors:** Ashkan H. Behzadi, Neil M. Khilnani, Weiguo Zhang, Amanda J. Bares, Srikanth R. Boddu, Robert J. Min, Martin R. Prince

**Affiliations:** 1 Department of Radiology, Weill Cornell Medical Center, 416 East 55th Street, New York, NY 10022 USA; 2 Meinig School of Biomedical Engineering, Cornell University, Ithaca, NY 14853 USA

**Keywords:** Magnetic resonance imaging, Deep venous thrombosis, Positioning, Hydration, Common femoral vein, Iliac vein

## Abstract

**Background:**

To determine the effect of hydration as well as prone versus supine positioning on the pelvic veins during cardiovascular magnetic resonance (CMR) venography.

**Methods:**

Under institutional review board approval, 8 healthy subjects were imaged with balanced steady state free precession, non-contrast CMR venography to measure common and external iliac vein volumes and common femoral vein cross-sectional area in the supine, prone and decubitus positions after dehydration and again following re-hydration. CMR venography from 23 patients imaged both supine and prone were retrospectively reviewed and measurements of common femoral and iliac veins areas were compared using Wilcoxon test.

**Results:**

Common femoral vein area on CMR venography increased with prone positioning (83 ± 35 mm^2^) compared to supine positioning (59 ± 21 mm^2^) (*p* = 0.02) and further increased with hydration to 123 ± 44 mm^2^ (*p* < 0.01). With right and left side down decubitus positioning, the common femoral vein area on dehydration increased from 29 ± 17 mm^2^ in the ante-dependent position to 134 ± 36 mm^2^ in the dependent position (*p* < 0. 001). Similarly, common and external iliac veins increased in volume with prone, 5.4 ± 1.9 cm^3^ and 5.8 ± 1.9 cm^3^ compared to supine positioning 4.6 ± 1.8 cm^3^ and 4.5 ± 1.9 cm^3^ (*p* = 0.01) and further increase with hydration to 6.7 ± 2.1 cm^3^ and 6.3 ± 1.9 cm^3^ (*p* = 0.01). CMR venography on patients also demonstrated an increase in mean common femoral vein luminal area from 103 ± 44 mm^2^ in supine position to 151 ± 52 mm^2^ with prone positioning (*p* < 0.001) as well as increases in common and external iliac vein volumes from 6.5 ± 2.6 cm^3^ and 8.0 ± 3.4 cm^3^ in the supine position to 7.5 ± 2.5 cm^3^ and 9.3 ± 3.6 cm^3^ with prone positioning (*p* < 0.01).

**Conclusions:**

Common femoral and common/external iliac vein size on CMR venography may be affected by position and hydration status. Routine clinical CMR venography of the pelvis could include prone positioning and avoiding dehydration to maximize pelvic vein distension.

**Electronic supplementary material:**

The online version of this article (10.1186/s12968-018-0503-6) contains supplementary material, which is available to authorized users.

## Background

Cardiovascular magnetic resonance (CMR) venography noninvasively assesses venous disease without ionizing radiation and often without requiring an exogenous contrast agent [1–4]. A recent systematic review and meta-analysis showed high sensitivity and specificity for CMR venography, comparable to iodinated contrast X-ray venography, for the detection of both acute and chronic deep venous thrombosis [5]. CMR venography may be especially useful in the pelvis of obese or pregnant patients where ultrasound is more challenging [5, 6].

**Fig. 1:**
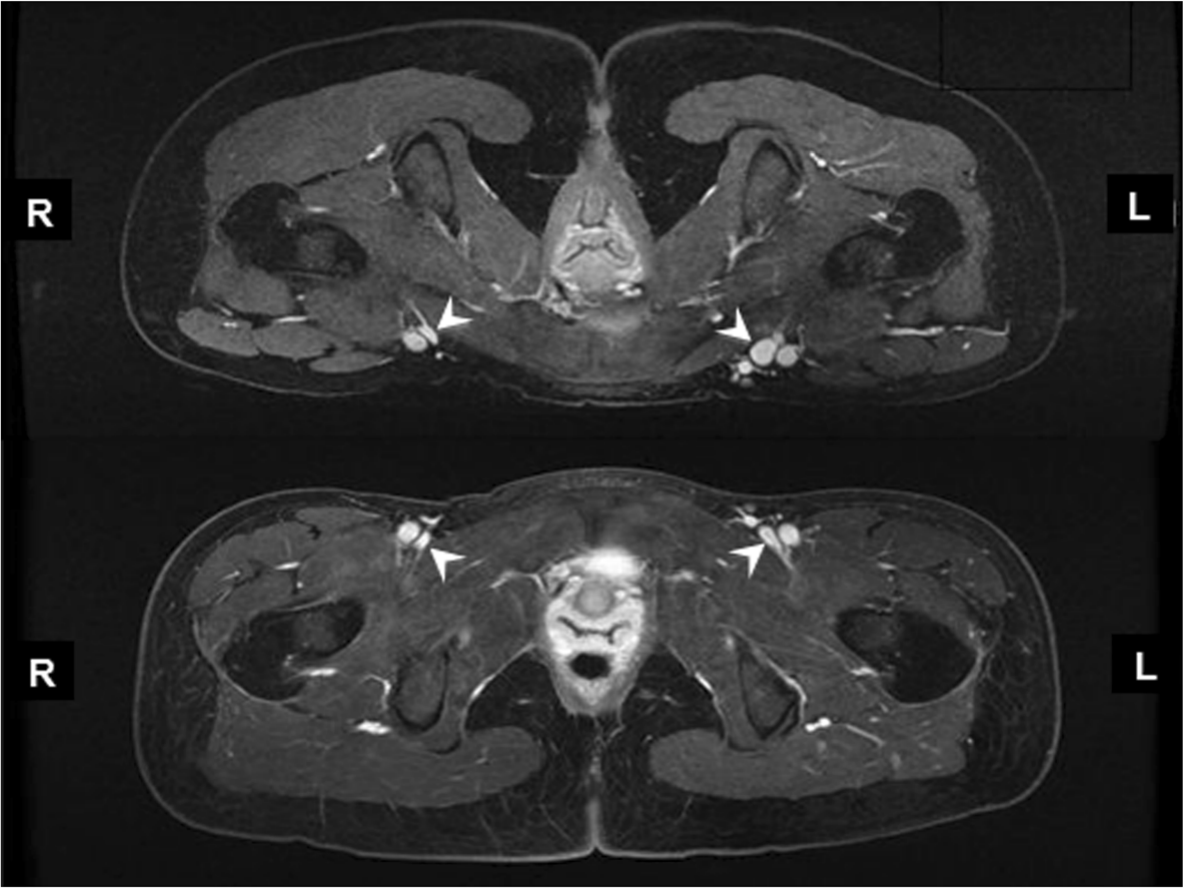
Patient transverse images of common femoral veins superior to arterial bifurcation. Common femoral veins appear large in the prone position (top pane) compared to veins in the supine position (bottom pane). Common femoral veins indicated by white arrowhead

However, when interpreting CMR images we have noticed that asymptomatic supine patients often have apparent common femoral vein narrowing. In one such patient, this apparent narrowing resolved with imaging in the prone position (Fig. 1). This patient had been instructed to arrive for her CMR venography examination fasting overnight (as per our routine at that time) and as a result, was likely dehydrated at the time of CMR scanning. After this patient, we changed our routine CMR venography of the pelvis to include both supine and prone balanced steady state free precession (bSSFP) images. In this paper, we present data from a prospective study on the effect of hydration status and scan position in healthy subjects as well as a retrospective review of patients undergoing CMR venography in both prone and supine positions to determine how to avoid imaging a collapsed common femoral vein, which might be mis-interpreted as narrowed on CMR images.

## Methods

### Prospective investigations in healthy volunteers

After institutional review board approval, 8 healthy subjects (5 women, 3 men) ranging in age from 22 to 24 years consented for this HIPPA compliant CMR venography study. Subjects signed informed consent, reported no health/medical issues and were instructed to avoid eating and drinking for a minimum of 6 h prior to CMR venography to ensure dehydration. Upon arrival to the scan facility, specific gravity urinalysis (using Siemens Multistix® 10 SG) was used to measure dehydration level. The threshold for clinical dehydration was a specific gravity of 1.025 [7]. Subjects underwent CMR venography of the pelvis using 2D bSSFP in prone, supine and both decubitus positions, and were then instructed to drink 1–2 L (water + Gatorade®) within 1 h to restore normal hydration levels. Urine specific gravity was tested again, and the bSSFP scanning protocol repeated.

### CMR scanning

All imaging was performed at 1.5 T (Signa HDx, GE Healthcare, Waukesha, Wisconsin, USA) using the body coil for signal transmission and a phased array coil for signal reception. Scanning positions included supine, right side down, left side down, and prone. In each position, pelvis and upper thigh veins were imaged in the transverse plane without intravenous contrast using bSSFP with the following scanning parameters: TR/TE/Flip angle = 4/1.3–1.7/45^o^ or 75^o^, FOV = 40 cm, slice thickness = 5 to 8 mm first 8 cases 8 mm and then 5 mm for the rest cases, bandwidth = 78.1–97.6 kHz, Matrix = 256 × 256 or 192 × 320. MR Venography images were not cardiac gated.

### Image analysis

Measurements were performed independently by two observers: one (AHB) with 2 years’ experience as an CMR Research fellow and the other (WZ) with 10 years’ experience as an abdominal imager, on a post-processing workstation (Advantage Windows 4.3, GE Healthcare). After calculating interobserver correlation coefficients, measurements from the two observers were averaged for subsequent analyses.

Common femoral vein cross-sectional area was measured immediately superior to the common femoral artery bifurcation (approximately at the center of the femoral head) where the imaging plane is perpendicular to the vein. These areas were measured by manually contouring a region of interest (ROI) around the vein lumen.

For the more tortuous iliac veins, it was not possible to find an image that was consistently perpendicular to those veins for both prone and supine positioning. So to evaluate the effect of position and hydration, iliac vein volumes were measured for an identical number of slices in prone and supine position beginning at common iliac bifurcation. This was performed on common and external iliac veins but was not possible for internal iliac veins which were often only reliably visualized on a few slices. Starting at the common iliac bifurcation and working inferiorly to measure external iliac vein volume and superiorly to measure common iliac vein volume, the lumen circumference was traced on consecutive slices using a program that automatically calculated volumes based on contours and slice spacing (3D paint, GE Advantage Windows Workstation, Milwaukee, Wisconsin, USA).

### Retrospective review of patient CMR venograms

Pelvic CMR venography was performed on 33 patients in both supine and prone positions for suspected deep venous thrombosis (*n* = 20), varicosities/venous insufficiency (*n* = 6), venous anomaly (*n* = 7). 10 patients with deep vein thrombosis were excluded leaving 23 cases with supine and prone imaging available for analysis (Table 1).

**Table 1:** Demographic data of MR venography patients

Patients’ Demographic Data	
Age (year)	50.4 (24–75)^a^
Gender(female/male)	16/17
Indications for MR Venography	
Varicosities/venous insufficiency	6
Suspected DVT	20
Suspected venous anomaly	7
Weight (kg)	82.5 ± 17.4

^a^range

Patients were scanned in supine and prone positions with the transverse 2D bSSFP sequence using the same parameters as listed for healthy subjects above. Common femoral vein luminal areas were measured independently by two observers blinded to all clinical information to measure interobserver variation. One reviewer with 2 years’ experience making CMR measurements as a CMR fellow measured common femoral vein luminal areas twice to assess intra observer variability. Observers manually contoured the common femoral vein perimeter on the transverse image aligning with the inferior edge of the femoral head as described above for the volunteers.

### Statistical analysis

Intra-class correlation coefficient (ICC) was used to investigate the reproducibility of continuous measurements [8]. The two-way ICC was computed for Interobserver ICCs and a one-way ICC was computed for the intra-observer ICCs. An ICC value of 1 indicates perfect agreement, ICC’s exceeding 0.7 are considered good and ICC’s exceeding 0.8 excellent, with observer error having a negligible effect on observed correlations between two (sets of) measurements [9]. After calculating ICCs, luminal area measurements of the two reviewers were averaged.

For the healthy subject group, differences in common femoral vein cross-sectional areas and common and external iliac veins volume between prone and supine positioning as well as dependent and ante-dependent decubitus positioning for common femoral vein while controlling for hydration status and between hydrated and dehydrated status while controlling for position were analyzed with non-parametric tests Wilcoxon test using Statistical software package SPSS Statistics^(^™^)^ V.23 (Statistical Package for the Social Sciences (SPSS), International Business Machines, Armonk, New York, USA).

The same statistical methodology has been applied for patients’ group differences in common femoral vein cross-sectional areas and common and external iliac veins volume between prone and supine positioning.

## Results

### Prospective investigations in healthy volunteers

All 8 healthy subjects had a urine specific gravity > 1.02 (lightly dehydrated), and 4 had urine specific gravity ≥ 1.03 (clinically dehydrated). Immediately following hydration with 1 to 2 L of water and Gatorade®, all healthy subjects had a urine specific gravity < 1.03, with a mean of 1.01 indicating successful rehydration.

Interobserver agreement for CMR venography measurements of common femoral vein area was excellent, ICC = 0.95 (95% CI 0.85–0.97). Intraobserver agreement was also excellent, ICC = 0.98 (95% CI 0.97–0.99). Similarly, interobserver agreement for common and external iliac vein volume measurements was excellent, ICC = 0.93 (95% CI 0.83–0.96). Intraobserver agreement was also excellent, ICC = 0.94 (95% CI 0.91–0.97).

**Fig. 2:**
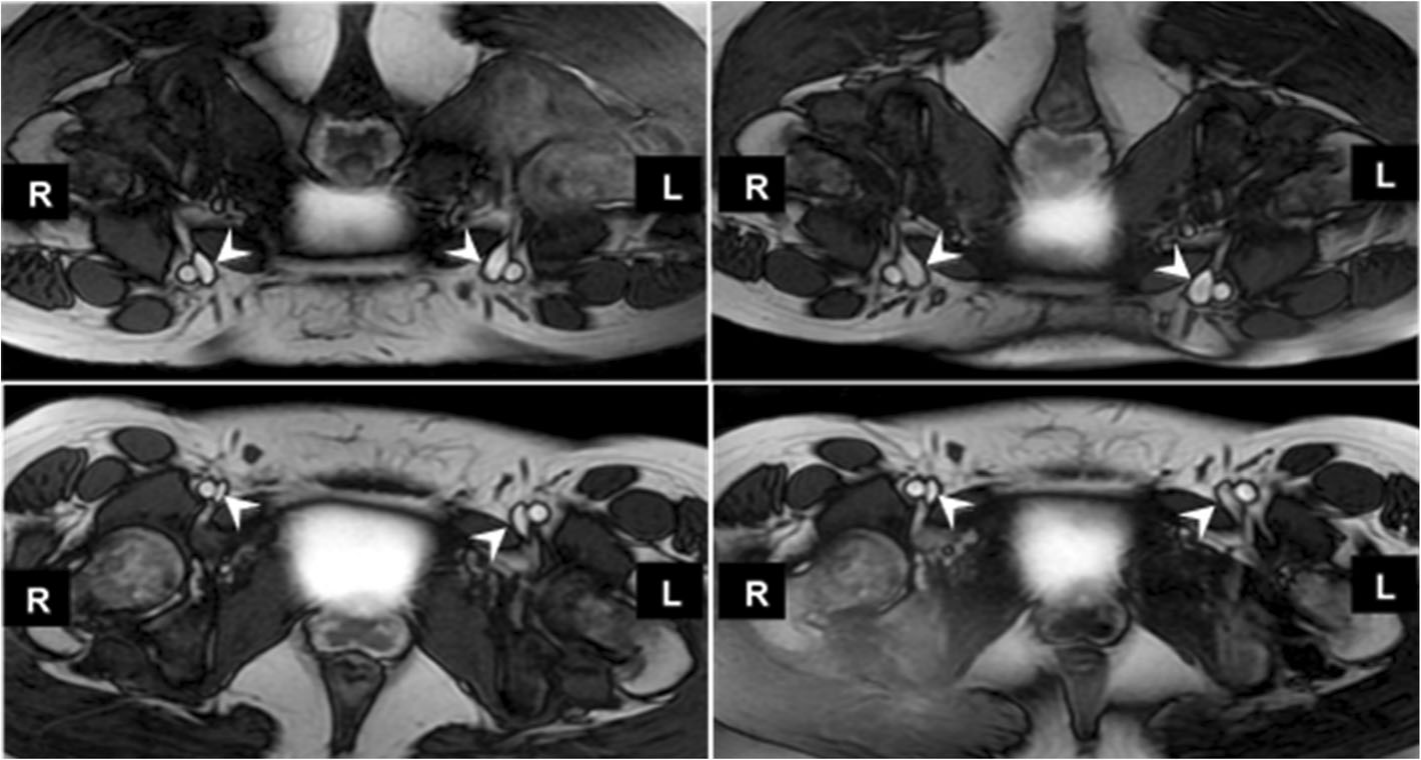
Prone vs. Supine scan positions in dehydrated and hydrated healthy subject. Common femoral veins (white arrows) appear larger in the prone position (top images) when compared to supine position (bottom images). Veins are noticeably smaller when the volunteer was dehydrated (left images) versus hydrated (right images)

Both prone positioning and hydration increased the common femoral vein area (see Table 2 and additional file 3). In dehydrated healthy subjects, common femoral vein area was increased 41% in the prone position (83 ± 35 mm^2^) compared to the supine position (59 ± 21 mm^2^) (*p* = 0.02). In the supine position, the common femoral vein area at dehydration (59 ± 21 mm^2^) increased 32% (to 78 ± 28 mm^2^) upon rehydration (*p* < 0.01). In the prone position, common femoral vein area increased 48% from (83 ± 35 mm^2^) during dehydration to (123 ± 44 mm^2^) upon hydration (*p* < 0.01) (Fig. 2).

**Table 2:** Common Femoral Vein Area (mm^2^) in supine vs. prone positioning and dehydration vs. hydration status in 8 volunteers

Patient Position	Common Femoral Vein Area (mm^2^)		
	Dehydrated	Hydrated	*P* value
Supine	59 ± 21	78 ± 28	0.001
Prone	83 ± 35	123 ± 44	0.002
*P* value	0.02	0.0007

**Fig. 3:**
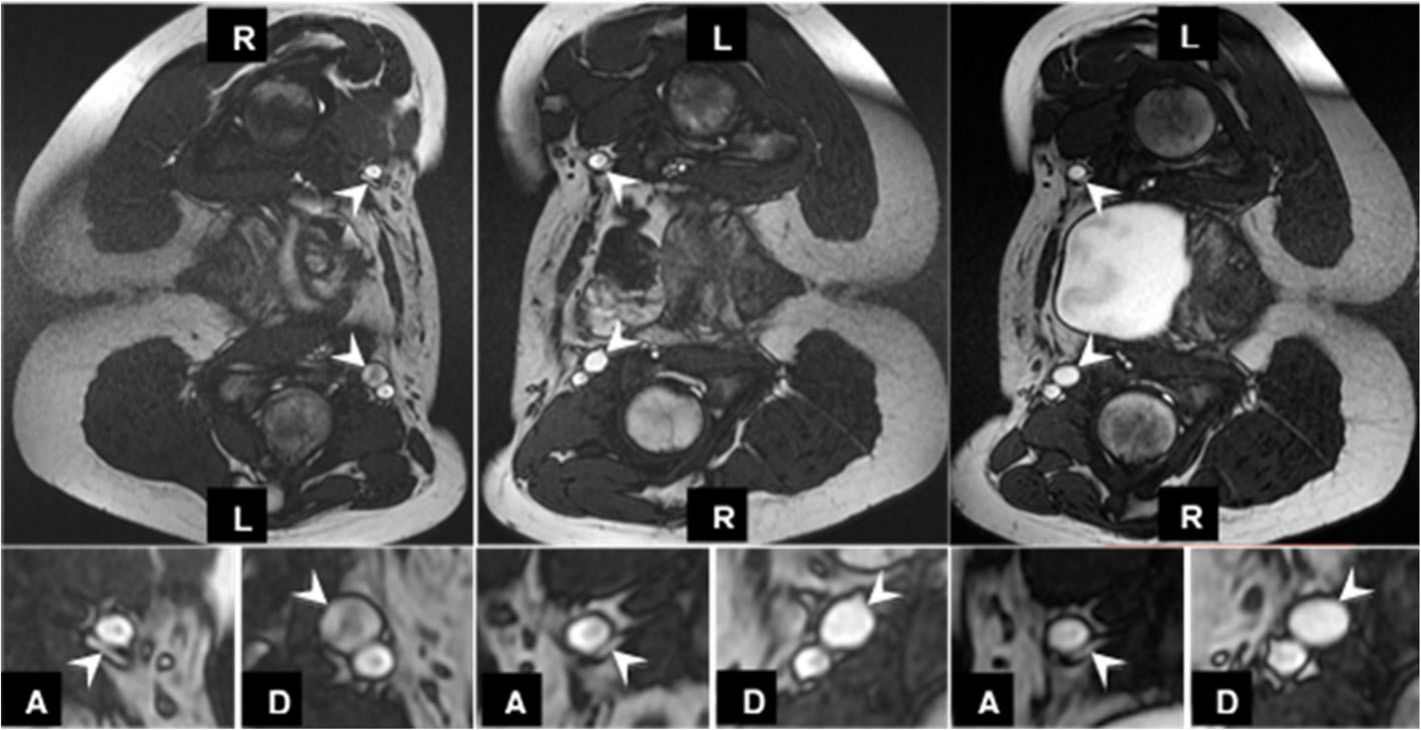
Left down and right down position in dehydrated and hydrated healthy subject. Common femoral veins (white arrows) appear significantly larger when dependent vs. anti-dependent in the volunteer in both left down (top left image) and right down (top middle image) positions. This is especially clear when magnified (bottom images; A = anti-dependent, D = dependent), where the anti-dependent vein appears nearly collapsed. Vein size did not appear larger when the subject was hydrated (right images)

In addition the common femoral vein area increased comparing dependent decubitus position to ante-dependent position in dehydrated (*p* < 0.01) and in hydrated (*p* < 0.01) healthy subjects (Fig. 3, Table 3 and additional file 3).

**Table 3:** Common Femoral Vein Area (mm^2^) in decubitus dependent vs. ante-decubitus positions for dehydration vs. hydration status in 8 volunteers

Decubitus Position	Common Femoral Vein Area (mm^2^)		
	Dehydrated	Hydrated	*P* value
Dependent	134 ± 36	135 ± 32	0.95
Ante-dependent	29 ± 17	36 ± 22	0.27
*P* value	< 0.0001	< 0.0001	

Both prone positioning and hydration also increased the common and external iliac vein volumes, (see Tables 2 and 3). The common and external iliac vein volumes were largest in prone hydrated subjects.

### Retrospective review of patient CMR venograms

Retrospective review of CMR venography on patients scanned both supine and prone demonstrated a 47% increase in mean common femoral vein luminal area from 103 ± 8 mm^2^ in the supine position to 151 ± 10 mm^2^ with prone positioning (*p* < 0.01) (Tables 4 and 5) (Fig. 1).

**Table 4:** Common Iliac Vein Volume (cm^3^) in supine vs. prone positioning with dehydration vs. hydration in 8 volunteers

Patient Position	Common Iliac Vein Volume (cm^3^)		
	Dehydrated	Hydrated	*P* value
Supine	4.6 ± 1.8	5.8 ± 2.1	<0.01
Prone	5.4 ± 1.9	6.7± 2.1	<0.01
*P* value	<0.001	<0.001

**Table 5:** External Iliac Vein Volume (cm^3^) in supine vs. prone positioning with dehydration vs. hydration in 8 volunteers

Patient Position	External Iliac Vein Volume (cm^3^)		
	Dehydrated	Hydrated	*P* value
Supine	4.5 ± 1.9	5.4 ± 2.0	<0.01
Prone	5.8 ± 1.9	6.3 ± 1.9	<0.01
*P* value	<0.01	<0.01	

**Fig. 4:**
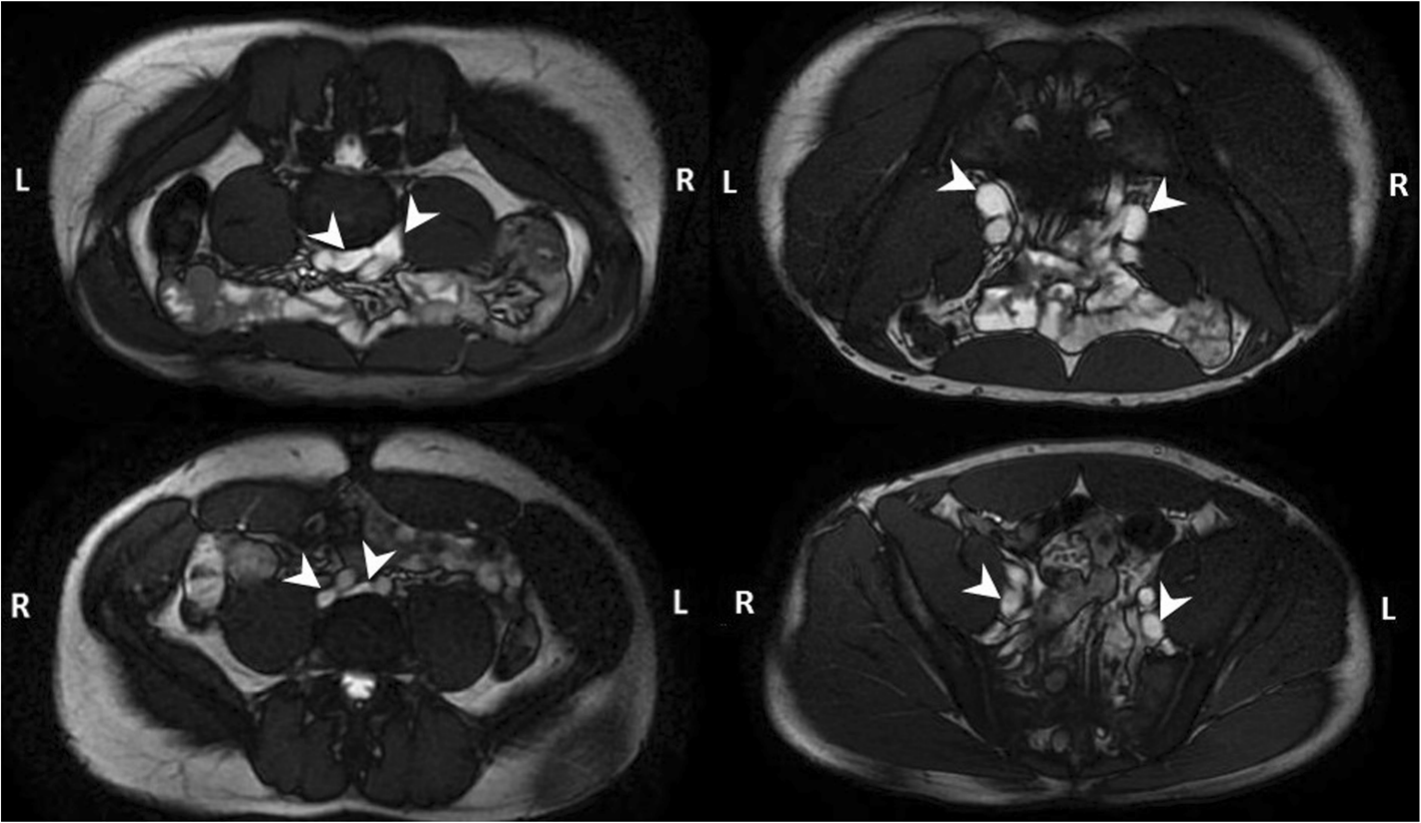
Prone hydrated (top) vs. Supine dehydrated (bottom) scan positions for common (left) and external (right) iliac veins in a healthy subject. Common iliac veins (white arrows) are larger in the hydrated/prone position (right, top image) when compared to dehydrated/supine position (right, bottom image). External iliac veins (right images, white arrows) are larger in the hydrated and prone position (top right) when compared to dehydrated and supine position (bottom right)

Common iliac vein volume increased 15% from 6.5 ± 2.6 cm^3^ in the supine position to 7.5 ± 2.5 cm^3^ with prone positioning (*p* < 0.01). Similarly external iliac vein volume increased 16% from 8.0 ± 3.4 cm^3^ in the supine position to 9.3 ± 3.6 cm^3^ with prone positioning (*p* < 0.01) (Tables 4 and 5) (Fig. 4).

This change in common femoral vein area and iliac vein volumes was not dependent on gender with similar results in males and females, (see Additional file 1: Table S1). This was also observed for May Thurner patients (see Additional file 2: Table S2) with a significant difference in common femoral vein areas (data from 5 cases) and a trend in common and external iliac vein volumes where data was only available from 4 subjects since one subject had a metallic stent.

## Discussion

CMR venography is commonly utilized in the pelvis where ultrasound can be limited, especially in obese patients where overlying fat and bowel gas artifact can adversely affect the ultrasound quality. When a vein appears narrowed on CMR venography, the possibility of prior deep venous thrombosis with recanalization and residual luminal narrowing must be considered, even when other signs of prior thrombosis are not present. However, data in 8 healthy subjects and 23 patients demonstrate that a normal common femoral or iliac vein may appear narrower in the supine position especially if the patient is dehydrated. Venous pathology may be excluded by switching to the dependent decubitus position or a prone position with hydration, both of which increase the degree of venous distention. Alternatively, in patients who can tolerate prone positioning, CMR venography can be performed in the prone position from the beginning. For advanced pregnancy where decubitus positioning is necessary, the side of any suspected venous obstruction should be dependent.

Positional effects on vein caliber are well known in the ultrasound literature [10–12]. Ultrasound has also shown that 15 degree Trendelenburg position [12, 13], gender [14, 15], age [15, 16] and ethnicity [15] can also affect vein diameter measurements. An ultrasound study of common femoral vein diameter for cannulation during hypovolemic states in newborn and infants showed a significant increase in femoral vein diameter with subjects in the reverse Trendelenburg position [16]. The change in diameter of the vein with the dependent decubitus position, and with reverse Trendelenburg and standing positions in veins is expected given the compliance of thin vein walls in normal conditions [17].

Common femoral vein areas on CMR venography in the prone and dependent decubitus positions resulted in values that are comparable to those found in the US literature [14, 18]. The CMR venography measured prone and decubitus common femoral vein area measurements are also similar to the reference values used to make estimates of percent stenosis on intravascular ultrasound and when making choices of stent sizes (12 mm diameter, 113 mm^2^) [19].

For many imaging studies including CMR scans, patients are encouraged to avoid eating or drinking prior to their appointment. This is an important requirement for tests examining intestinal peristalsis or to minimize the potential volume of emesis and risk of aspiration with contrast agent injection induced nausea. However, dehydration may have a detrimental effect on scans specifically examining blood vessel volume/area. Because vein diameter or distension is dependent on blood volume, dehydration reduces vein size, particularly the ante-dependent veins.

In our healthy subject studies, dehydrated subjects demonstrated a significantly smaller common femoral vein area compared to imaging the same subjects following 1 to 2 L of fluid hydration. In this work we were able to confirm hydration status with use of urine dipsticks. These dipsticks may also be useful for confirming that patients undergoing CMR venography are adequately hydrated. With urine specific gravity in excess of 1.02, some healthy subjects reported symptoms of dehydration including fatigue, thirst, headaches, and dark yellow urine.

One limitation of this paper is the small number of healthy subjects and patients. Another limitation is the use of bSSFP instead of time-of-flight or gadolinium enhanced CMR venography for the normal subjects and patients as bSSFP may have more artifacts and can be less sensitive and less specific for deep venous thrombosis. However, bSSFP has high SNR for quickly producing high resolution images over large volumes and in these volunteers, deep venous thrombosis was not suspected. Since time-of-flight is based upon in-flow, positional effects on in-flow limit the ability to generalize these results to time-of-flight sequences. Yet another limitation is that volunteers were young and healthy and not age or co-morbidity matched to the patients. Also there are no data on blood pressure or other risk factors for vascular disease.

## Conclusion

Patients should be encouraged to hydrate before arriving for CMR venography to ensure veins are fully distended. In addition, CMR technicians should consider performing CMR venography of the pelvis in the prone position or decubitus position with the symptomatic leg down. If iliac or femoral veins appear stenotic with supine imaging, changing to prone or decubitus positioning may help to more fully evaluate if venous disease is actually present and not an artifact of scanning position.

## Additional files

Additional file 1: **Table S1a.** Right and left common femoral vein area (mm^2^) in supine vs. prone positioning in male patients undergoing CMR venography. **Table S1b.** Right and left common iliac vein volume (cm^3^) in supine vs. prone positioning in male patients undergoing CMR venography. **Table S1c.** Right and left common femoral vein area (cm^3^) in supine vs. prone positioning in male patients undergoing CMR venography. (DOCX 16 kb)

Additional file 2: **Table S2a.** Right and left common femoral vein area (mm^2^) in supine vs. prone positioning in 5 May-Thurner syndrome patients undergoing CMR venography. **Table S2b.** Right and left common iliac vein volume (cm^3^) in supine vs. prone positioning in 5 May-Thurner syndrome patients undergoing CMR venography. **Table S2c.** Right and left common femoral vein area (cm^3^) in supine vs. prone positioning in 5 May-Thurner syndrome patients undergoing CMR venography. (DOCX 16 kb)

Additional file 3: **Table S3a.** Common femoral vein area normalized by weight (mm^2^/kg) in supine vs. prone positioning and dehydration vs. hydration status in 8 healthy subjects. **Table S3b.** Common femoral vein area normalized by weight (mm^2^/kg) in decubitus dependent vs. ante-decubitus positions for dehydration vs. hydration status in 8 healthy subjects. **Table S3c.** Common iliac vein volume normalized by weight (mm^3^/kg) in supine vs. prone positioning with dehydration vs. hydration in 8 healthy subjects. **Table S3d.** External iliac vein volume normalized by weight (mm^3^/kg) in supine vs. prone positioning with dehydration vs. hydration in 8 healthy subjects. (DOCX 21 kb)

## Abbreviations

bSSFP: Balanced steady-state free precession
CFV: Common femoral vein
CMR: Cardiovascular magnetic resonance
FOV: Field of view
ICC: Intra-Class Correlation Coefficient
ROI: Region of interest
SNR: Signal-to-noise ratio
TE: Echo time
TR: Repetition time
