# Supplementary material for: Pelvic cardiovascular magnetic resonance venography: venous changes with patient position and hydration status
Source: J Cardiovasc Magn Reson. 2019 Jan 3;21:3. doi: 10.1186/s12968-018-0503-6 (PMC6317255; doi:10.1186/s12968-018-0503-6)
Supplement: Supplementary file 1 — Table S1a. Right and left common femoral vein area (mm2) in supine vs. prone positioning in male patients undergoing CMR venography. Table S1b. Right and left common iliac vein volume (cm3) in supine vs. prone positioning in male patients undergoing CMR venography. Table S1c. Right and left common femoral vein area (cm3) in supine vs. prone positioning in male patients undergoing CMR venography. (DOCX 16 kb) [file 12968_2018_503_MOESM1_ESM.docx]

**Additional file 1: Table S1a.** Right and left Common Femoral Vein Area (mm^2^) in supine vs. prone positioning in male patients undergoing MR Venography.

|  | **Common femoral Vein Area (mm^2^)** | |
| --- | --- | --- |
| **Patient Position** | **Right** | **left** |
| **Supine(male)** | 107.79±44.3 | 104.7±44.1 |
| **Prone(male)** | 155.1±49.1 | 156.4±46.1 |
| ***P value*** | 0.004 | 0.003 |
| **Supine(female)** | 97.7±38.3 | 81.2±33.7 |
| **Prone(female)** | 137.3±61.3 | 130.2±51.4 |
| ***P value*** | 0.004 | 0.003 |

**Additional file 1: Table S1b.** Right and left Common Iliac Vein volume (cm^3^) in supine vs. prone positioning in male patients undergoing MR Venography.

|  | **Common iliac** **Vein volume (cm^3^)** | |
| --- | --- | --- |
| **Patient Position** | **Right** | **left** |
| **Supine(male)** | 5.8±3.0 | 7.4±2.9 |
| **Prone(male)** | 6.7±3.0 | 8.6±3.5 |
| ***P value*** | 0.05 | 0.12 |
| **Supine(female)** | 6.6± 1.54 | 7.9±1.8 |
| **Prone(female)** | 7.6±1.3 | 9.3±2.2 |
| ***P value*** | 0.004 | 0.03 |

**Additional file 1: Table S1c.** Right and left Common Femoral Vein Area (cm^3^) in supine vs. prone positioning in male patients undergoing MR Venography.

|  | **External iliac Vein volume (cm^3^)** | |
| --- | --- | --- |
| **Patient Position** | **Right** | **left** |
| **Supine(male)** | 8.1±4.0 | 8.8±4.4 |
| **Prone(male)** | 9.6±4.9 | 10.3±4.3 |
| ***P value*** | 0.01 | 0.01 |
| **Supine(female)** | 7.5±1.8 | 8.12±1.6 |
| **Prone(female)** | 8.7±0.6 | 9.2±1.1 |
| ***P value*** | 0.01 | 0.01 |
