# Supplementary material for: Pelvic cardiovascular magnetic resonance venography: venous changes with patient position and hydration status
Source: J Cardiovasc Magn Reson. 2019 Jan 3;21:3. doi: 10.1186/s12968-018-0503-6 (PMC6317255; doi:10.1186/s12968-018-0503-6)
Supplement: Supplementary file 2 — Table S2a. Right and left common femoral vein area (mm2) in supine vs. prone positioning in 5 May-Thurner syndrome patients undergoing CMR venography. Table S2b. Right and left common iliac vein volume (cm3) in supine vs. prone positioning in 5 May-Thurner syndrome patients undergoing CMR venography. Table S2c. Right and left common femoral vein area (cm3) in supine vs. prone positioning in 5 May-Thurner syndrome patients undergoing CMR venography. (DOCX 16 kb) [file 12968_2018_503_MOESM2_ESM.docx]

**Additional file 2: Table S2a.** Right and left Common Femoral Vein Area (mm^2^) in supine vs. prone positioning in 5 May-Thurner syndrome patients undergoing MR Venography.

|  | **Common Femoral Vein Area (mm^2^)** | |
| --- | --- | --- |
| **Patient Position** | **Right** | **left** |
| **Supine** | ^107.5± 47.3^ | ^98.8± 48.1^ |
| **Prone** | ^143.3± 40.0^ | ^117.8± 54.4^ |
| **P-value** | ^0.04^ | ^0.04^ |

**Additional file 2: Table S2b.** Right and left Common Iliac Vein volume (cm^3^) in supine vs. prone positioning in 5 May-Thurner syndrome patients undergoing MR Venography.

|  | **Common iliac Vein volume (cm^3^)** | |
| --- | --- | --- |
| **Patient Position** | **Right** | **left** |
| **Supine** | ^6.8± 2.7^ | ^9.2±1.5^ |
| **Prone** | ^8.2± 2.4^ | ^10.8±2.0^ |
| **P-value** | ^0.068^ | ^0.068^ |

**Additional file 2: Table S2c.** Right and left Common Femoral Vein Area (cm^3^) in supine vs. prone positioning in 5 May-Thurner syndrome patients undergoing MR Venography.

|  | **External iliac Vein Area (cm^3^)** | |
| --- | --- | --- |
| **Patient Position** | **Right** | **left** |
| **Supine** | ^9.0±2.64^ | ^9.7±3.0^ |
| **Prone** | ^11.1±3.2^ | ^11.2±3.3^ |
| **P-value** | ^0.068^ | ^0.068^ |
