# Supplementary material for: Pelvic cardiovascular magnetic resonance venography: venous changes with patient position and hydration status
Source: J Cardiovasc Magn Reson. 2019 Jan 3;21:3. doi: 10.1186/s12968-018-0503-6 (PMC6317255; doi:10.1186/s12968-018-0503-6)
Supplement: Supplementary file 3 — Table S3a. Common femoral vein area normalized by weight (mm2/kg) in supine vs. prone positioning and dehydration vs. hydration status in 8 healthy subjects. Table S3b. Common femoral vein area normalized by weight (mm2/kg) in decubitus dependent vs. ante-decubitus positions for dehydration vs. hydration status in 8 healthy subjects. Table S3c. Common iliac vein volume normalized by weight (mm3/kg) in supine vs. prone positioning with dehydration vs. hydration in 8 healthy subjects. Table S3d. External iliac vein volume normalized by weight (mm3/kg) in supine vs. prone positioning with dehydration vs. hydration in 8 healthy subjects. (DOCX 21 kb) [file 12968_2018_503_MOESM3_ESM.docx]

**Table 3a.** Common Femoral Vein Area normalized by weight (mm^2^/kg) in supine vs. prone positioning and dehydration vs. hydration status in 8 volunteers.

|  | **Weight Adjusted Common Femoral Vein Area (mm^2^/kg)** | |  |
| --- | --- | --- | --- |
| **Patient Position** | **Dehydrated** | **Hydrated** | **P value** |
| **Supine** | 0.73± 0.30 | 0.96 ± 0.29 | 0.001 |
| **Prone** | 1 ± 0.25 | 1.45 ± 0.41 | 0.002 |
| **P value** | 0.02 | 0.001 |  |

**Table 3b.** Common Femoral Vein Area normalized by weight (mm^2^/kg) in decubitus dependent vs. ante-decubitus positions for dehydration vs. hydration status in 8 volunteers.

|  | **Weight Adjusted Common Femoral Vein Area (mm^2^/kg)** | |  |
| --- | --- | --- | --- |
| **Decubitus Position** | **Dehydrated** | **Hydrated** | **P value** |
| **Dependent** | 1.66± 0.40 | 1.65 ± 0.35 | 0.95 |
| **Ante-dependent** | 0.33± 0.15 | 0.42 ± 0.27 | 0.27 |
| **P value** | <0.0001 | <0.0001 |  |

**Table 3c.** Common Iliac Vein Volume normalized by weight (mm^3^/kg) in supine vs. prone positioning with dehydration vs. hydration in 8 volunteers.

|  | **Weight Adjusted Common Iliac Vein Volume (** **mm^3^/kg )** | |  |
| --- | --- | --- | --- |
| **Position** | **Dehydrated** | **Hydrated** | **P value** |
| **Supine** | 67 ± 28 | 86 ± 40 | <0.01 |
| **Prone** | 79 ± 29 | 98 ± 44 | <0.01 |
| **P value** | <0.001 | <0.001 |  |

**Table 3d.** External Iliac Vein Volume normalized by weight (mm^3^/kg) in supine vs. prone positioning with dehydration vs. hydration in 8 volunteers.

|  | **Weight Adjusted External Iliac Vein Volume (** **mm^3^/kg )** | |  |
| --- | --- | --- | --- |
| **Patient Position** | **Dehydrated** | **Hydrated** | **P value** |
| **Supine** | 48.2 ± 17.0 | 87.0 ± 33.4 | <0.01 |
| **Prone** | 81.0 ± 29.0 | 95.0 ± 34.2 | <0.01 |
| **P value** | <0.01 | <0.01 |  |
